# Supplementary material for: Improved production of Taxol® precursors in S. cerevisiae using combinatorial in silico design and metabolic engineering
Source: Microb Cell Fact. 2023 Nov 29;22:243. doi: 10.1186/s12934-023-02251-7 (PMC10687855; doi:10.1186/s12934-023-02251-7)
Supplement: Supplementary file 1 — Additional file 1: Table S1. The primer list used to produce the donor DNA parts for the gene deletions. Table S2. crRNA sequences used to target the corresponding genes for deletions. Table S3. The primer list used to produce the donor DNA parts for the genomic integrations. Table S4. The yeast strains used in the study. Table S5. The gene candidates predicted by the design algorithms. Table S6. The components of the yeast nitrogen base used in the study. Table S7. The components of the complete supplement mixture used in the study. Figure S1. The ACtivE method for gene deletion. Figure S2. The ACtivE method for genomic integration. Figure S3. Acetyl-CoA-centred sub-metabolite interaction map of the wild-type model. Figure S4. Acetyl-CoA-centred sub-metabolite interaction map of DTR1 deleted model. Figure S5. Acetyl-CoA-centred sub-metabolite interaction map of ACH1 deleted model. Figure S6. Acetyl-CoA-centred sub-metabolite interaction map of MLS1 deleted model. Figure S7. Acetyl-CoA-centred sub-metabolite interaction map of DIT1 deleted model. Figure S8. Acetyl-CoA-centred sub-metabolite interaction map of LPP1 deleted model. Figure S9. Acetyl-CoA-centred sub-metabolite interaction map of ISC1 deleted model. Figure S10. de novo purine nucleotide biosynthesis pathway. Figure S11. Phosphopantothenate biosynthesis pathway. Figure S12. Dissolved oxygen (DO) concentrations of three-day cultures of KM1-derived strains measured by the BioLector microbioreactor system in the galactose-containing CSM. Figure S13. Dissolved oxygen (DO) concentrations of three-day cultures of EJ1-derived strains measured by the BioLector microbioreactor system in the glucose-containing CSM. Figure S14. Gas chromatography shows the compounds’ peaks produced by KM32 and the mass spectrum of taxadiene. Figure S15. Gas chromatography shows the compounds’ peaks produced by KM32 and the mass spectrum of T5α-ol. The retention time of the T5α-ol peak was at 8.20th minutes. Figure S16. Gas chromatog [file 12934_2023_2251_MOESM1_ESM.pdf]

Additional File 1

Improved Production of Taxol® Precursors in *S. cerevisiae* using Combinatorial *in silico* Design and Metabolic Engineering

Koray Malcı [a,b,1,*], Rodrigo Santibáñez [c], Nestor Jonguitud-Borrego [a,b], Jorge H. Santoyo-Garcia [a,b], Eduard J. Kerkhoven [d,e,f], Leonardo Rios-Solis [a,b,g,h,*]

[a] Institute for Bioengineering, School of Engineering, University of Edinburgh, King's Buildings, Edinburgh EH9 3BF, United Kingdom

[b] Centre for Engineering Biology, University of Edinburgh, King's Buildings, Edinburgh EH9 3BF, United Kingdom

[c] Department of Pediatrics, University of California, San Diego, 9500 Gilman Drive, La Jolla, CA, 92093-0760, USA

[d] Department of Life Sciences, Chalmers University of Technology, Kemivägen 10, SE412 96, Gothenburg, Sweden

[e] SciLifeLab, Chalmers University of Technology, SE-412 96, Gothenburg, Sweden

[f] Novo Nordisk Foundation Center for Biosustainability, Technical University of Denmark, DK-2800 Kgs. Lyngby, Denmark

[g] School of Natural and Environmental Sciences, Molecular Biology and Biotechnology Division, Newcastle University, Newcastle upon Tyne NE1 7RU, United Kingdom

[h] Department of Biochemical Engineering, The Advanced Centre for Biochemical Engineering, University College London, Gower Street, London, WC1E 6BT, UK

[1] Current address: Department of Bioengineering, Imperial College London, London, SW7 2AZ, United Kingdom

* Corresponding authors: Dr Leonardo Rios Solis (leo.rios@ucl.ac.uk), Dr Koray Malcı (k.malci@imperial.ac.uk)

in silico Design and Analyses

Our methodology utilized three design algorithms (OptKnock, OptGene, and OptForce) in conjunction with the latest yeast genome scale model 8.5.0. We conducted simulations under complete synthetic yeast medium conditions, using either glucose or galactose as carbon sources, and targeted overproductions of cytosolic acetyl-CoA or GGPP.

OptKnock identifies reaction knock-outs to enhance target metabolism [1], while OptForce can predict reaction upregulations, downregulations, or knock-outs [2]. On the other hand, OptGene suggests gene deletions for optimising target compound production [3]. When OptKnock and OptForce suggested reactions, we considered the associated genes for those reactions.

These algorithms can also suggest a set of genomic modifications. However, we adopted a combinatorial approach in this study. We pooled predicted genes (or corresponding genes of reactions) based on specific criteria. Reactions not associated with any genes were excluded from further analysis. For instance, OptKnock suggested deleting reaction r_4362 (a dipeptidase reaction converting Gly-Met to L-methionine and L-glycine in vacuole) and r_4483 (glycine transport from cytosol to vacuole) to improve GGPP production in glucose-containing medium. However, these reactions lack associated genes in the model, so we disregarded such predictions for wet-lab validation.

To streamline the labour and cost involved, we eliminated reactions associated with multiple genes. For example, OptKnock recommended knocking out r_0832 for GGPP overproduction in glucose-containing medium. However, r_0832 involves three genes (YDR148C, YFL018C, and YIL125W) for knockout, so we focused on reactions associated with single genes.

Furthermore, we did not consider reaction downregulations suggested by the OptForce framework, as fine-tuning reaction rates might require alternative strategies like enzymatic inhibitions or gene regulations.

To combine modifications from different frameworks, we shortlisted the most promising candidates through further analysis. We determined maximum potential fluxes using flux variability analysis (FVA) algorithm [4] and assessed flux improvements by constructing a metabolite interaction network [5, 6]. Subsequently, we experimentally evaluated 17 genomic modifications, consisting of nine gene deletions and eight gene overexpressions. The second-level strains were engineered based on the experimental findings.

In our simulation, the inorganic components of the complete synthetic medium (CSM) were left unconstrained, while the uptake rates of carbon sources—glucose or galactose—were maintained at a constant level, as these are potential limiting factors in the growth medium. Specifically, we allowed unconstrained uptake of the inorganic compounds listed in Table S6, which include phosphate, sulfate, ammonium, oxygen, sodium, potassium, chloride, copper, manganese, zinc, magnesium, calcium, iron, and hydrogen.

Conversely, we enabled waste removal pathways for acetate, carbon dioxide, ethanol, glycolaldehyde, diphosphate, water, glycerol, and acetaldehyde secretion during the simulations.

Table S1: The primer list used to produce the donor DNA parts for the gene deletions

Name	Sequence 5' to 3'
DPP1 UHA For	CAAGTTCGTTGCACTGTATTTC
DPP1 UHA Rev + DHA	AGAATCAGAATTAAATCATAGCAAACGACCCCACATGACATACGAAATATACG
DPP1 DHA For + UHA	ATAAATACGTATATTTCGTATGTCATGTGGGGTCGTTTGCTATGATTTAATTC
DPP1 DHA Rev	GTATCAGTCACAGGTACGG
DPP1 Col Rev	CCATTGATGATCCTCTTCCG
OAR1 UHA For	CTTCAACATCATCGTCACC
OAR1 UHA Rev + DHA	TGGTATAGACGTGGGAGAAGAAAAGTCTGGCAGTGAACACATTTTCACATC
OAR1 DHA For + UHA	AAGAAACGTGATGTGAAAATGTGTTCACTGCCAGACTTTTCTTCTCCCAC
OAR1 Col Rev	GATTTACAGAAGTTTTAGCTGC
MDH3 UHA For	CAACACATCGGTCATAGTAG
MDH3 UHA Rev + DHA	CTTCGTAAATCAAGGGAAAACACTTGTCAGGAAGGAAAGGAAACCATATCC
MDH3 DHA For + UHA	CCACTAGGAGGATATGGTTTCCTTTCCTTCCTGACAAGTGTTTTCCCTTG
MDH3 DHA Rev	CTCTCAGTTGATTTTCCGTG
MDH3 Col Rev	GTATCCATCGATACCAGTGT
ACH1 UHA For	CATATGACATACGTATTAGCCGC
ACH1 UHA Rev + DHA	ATAAATATGCAAGAAAAAACAACGCATTGGGTTTGTTTTGCCGCTATTGTC
ACH1 DHA For + UHA	GTCTTACTAGACAATAGCGGCAAAACAAACCCAATGCGTTGTTTTTTCTTG
ACH1 DHA Rev	CACCACTGATAGATAACACACA
ACH1 Col Rev	GAGTACCCACTCCTGTAAAAC
MLS1 UHA For	CAGTATTACCCTACATTGCTATC
MLS1 UHA R + DHA	AGATGATTCATTGCTAACTACGAAACGAAGGTGCTTTTACTACTTTGTTTAGTTC
MLS1 DHA For + UHA	GTTTTGAACTAAACAAAGTAGTAAAAGCACCTTCGTTTCGTAGTTAGCAATG
MLS1 DHA Rev	CACACTCATATGTGAGTTAGATG
MLS1 Col Rev	GGTAAGAATATCTCCCACTGTAG
DIT1 UHA For	GAGTCCCTGGAAGGAAAATTATTG
DIT1 UHA Rev + DHA	TATTCCCCCTCTGTTAAATGGAATTGTGTGGCGGAGGAGCACAATTTATG
DIT1 DHA For + UHA	TAAATTTTCACATAAATTGTGCTCCTCCGCCACACAATTCCATTTAACAGAGG
DIT1 DHA Rev	CTGATGCCTCAAGATTTAACC

DIT Col Rev	GATGAAGAAAATGAAGAGGCAGG
LPP1 UHA For	CACCGACGGATTCAGAG
LPP1 UHA Rev + DHA	CATCAACGCCTAAGGAAACTCGTCATATTCCACTTACAGAGTCCTATCAGG
LPP1 DHA For + UHA	TTATTCTTTCCTGATAGGACTCTGTAAGTGGAATATGACGAGTTTCCTTAGG
LPP1 DHA Rev	CTATATGAAAACTTCACAGGGAG
LPP1 Col Rev	GTCTCTGGAGCTGTTCTAG
ISC1 UHA For	CGTAATTGGACACACTCTTTAC
ISC1 UHA Rev + DHA	TTCTCCGTGATTGCTTTGCATCTATTGACGCTCGAAGATTTAAGCCAAACC
ISC1 DHA For + UHA	ATTTATTTTGGTTTGGCTTAAATCTTCGAGCGTCAATAGATGCAAAGCAATC
ISC1 DHA Rev	GACATTTACTTAATGTGGAGCAC
ISC1 Col Rev	CTTCAAACTCAGATTGACCATC
DTR1 UHA For	GTTGCTATGTTCGGATGTAC
DTR1 UHA Rev + DHA	TTTCTTGAAGTCCTTGGGATGAGTGATGAGCAACAACAACTTTTCTTACTACC
DTR1 DHA For + UHA	GAAGGATGGTAGTAAGAAAAGTTGTTGTTGCTCATCACTCATCCCAAGGAC
DTR1 DHA Rev	GGTCTAACTGACACTACTGTTCC

* The **red** sequences show the overlapping fragments with neighbour parts, while the **black** sequences show the annealing fragments.
** Plus (+) sign shows the overlapping neighbour parts targeted by the **red** sequences
*** If the overlapping sequences (**red**) show higher affinity than the annealing sequences (**black**) for the same DNA template, only annealing parts should be used first to amplify the target regions.
**** For: forward, Rev: reverse, UHA: upstream homology arm, DHA: downstream homology arm, Col: for colony PCR (coupled with corresponding UHA For)

Table S2: crRNA sequences used to target the corresponding genes for deletions

Target Gene	Sequence 5' – 3'	Chromosome
DPP1	GCAGTAAATAAAGTGTCCAA *TGG*	IV
OAR1	AGGAGATGGCCGGAACTCAG *TGG*	XI
MDH3	TGTTATTGGGGGTCATTCAG *GGG*	IV
ACH1	CTGAAAGTGGACGACAAGTG *TGG*	II
MLS1	AAGATTACATTGGGATCCCA *AGG*	XIV
DIT1	CCTTGTCAAGATATATCCAC *CGG*	IV
LPP1	ATGAAACTTGAATGTCCGCT *TGG*	IV
ISC1	ACTCCTGGGAGCAATTGCAT *GGG*	V
DTR1	GGTAATGGAGACCCTAAATG *GGG*	II

* The **red** nucleotides represent the corresponding PAM sequences

Table S3: The primer list used to produce the donor DNA parts for the genomic integrations

Name	Sequence 5' to 3'
UHA 1603 For	GGCTATGGTGGTGATGTCTG
DHA 1603 Rev	CTGTCTCCGCTATGTCAGTTAC
UHA 1603 Rev + pGAL1	CTAATCCGTACTTCAATATAGCAATGAGCGAGGAAAAAAACAGTTGTACATTGG
pGAL For + UHA 1603	GTTACCAATGTACAACTGTTTTTTTCCTCGCTCATTGCTATATTGAAGTAC
pGAL Rev + ILV2	GAAGTTTTTTAGCGTAGATTGTCTGATCATTATAGTTTTTTCTCCTTGACG
ILV2 For + pGAL	ATACTTTAACGTCAAGGAGAAAAAACTATAATGATCAGACAATCTACGCTAAAAAAC
ILV2 Rev + DHA 1603	AAAAGCTCGTGAATACGGCAAGAACGAAGCGCGGTTAAATTCGTATTGGCC
DHA 1603 For + ILV2	AAGAACAGTGGCCAATACGAATTTAACCGCGCTTCGTTCTTGCCGTATTC
pGAL Rev + TRR1	ACCAATGATAGTAACTTTGTTGTGAACCATTATAGTTTTTTCTCCTTGACG
TRR1 For + pGAL	ATACTTTAACGTCAAGGAGAAAAAACTATAATGGTTCACAACAAAGTTACTATC
TRR1 Rev + DHA 1603	AAAAGCTCGTGAATACGGCAAGAACGAAGCGTCAGTAGCTGTAATATCAGATG
DHA 1603 For + TRR1	AATAAAGCATCTGATATTACAGCTACTGACGCTTCGTTCTTGCCGTATTC
pGAL Rev + ADE4	TGCTAATACAATACCTAAAATACCACACATTATAGTTTTTTCTCCTTGACG
ADE4 For + pGAL	ATACTTTAACGTCAAGGAGAAAAAACTATAATGTGTGGTATTTTAGGTATTG
ADE4 Rev + DHA 1603	AAAAGCTCGTGAATACGGCAAGAACGAAGCCTTTTCTTTTTGTACAAGCTG
DHA 1603 For + ADE4	AGCAATCCGCAGCTTGTACAAAAAGAAAAGGCTTCGTTCTTGCCGTATTC
pGAL Rev + ADE57	ACCGTTTCCTAAAACGAGAATGTTGAGCATTATAGTTTTTTCTCCTTGACG
ADE57 For + pGAL	ATACTTTAACGTCAAGGAGAAAAAACTATAATGCTCAACATTCTCGTTTTAGG
ADE57 Rev + DHA 1603	AAAAGCTCGTGAATACGGCAAGAACGAAGCCTACAACTCCTCAACAGTATTCTC
DHA 1603 For + ADE57	TAGCAAGAGAATACTGTTGAGGAGTTGTAGGCTTCGTTCTTGCCGTATTC
pGAL Rev + ADE13	TGGCGTAGTGTAATTGTCGTAGTCAGGCATTATAGTTTTTTCTCCTTGACG
ADE13 For + pGAL	ATACTTTAACGTCAAGGAGAAAAAACTATAATGCCTGACTACGACAATTAC
ADE13 Rev + DHA 1603	AAAAGCTCGTGAATACGGCAAGAACGAAGCCGACTGAGTAAGAACCGTTTC
DHA 1603 For + ADE13	TAAAAATATGAAACGGTTCTTACTCAGTCGGCTTCGTTCTTGCCGTATTC
pGAL Rev + ECM31	GGTGCATAATTGTCTTTTCATTATATTCATTATAGTTTTTTCTCCTTGACG
ECM31 For + pGAL	ATACTTTAACGTCAAGGAGAAAAAACTATAATGAATATAATGAAAAGACAATTATGC
ECM31 Rev + DHA 1603	AAAAGCTCGTGAATACGGCAAGAACGAAGCGATAAAGACAACAACTCGTG
DHA 1603 For + ECM31	CTTGGTAGCCCACGAGTTGTTGTCTTTATCGCTTCGTTCTTGCCGTATTC
pGAL Rev + CAB1	GTAAGATATCTCTTGAGTAATTCGCGGCATTATAGTTTTTTCTCCTTGACG
CAB1 For + pGAL	ATACTTTAACGTCAAGGAGAAAAAACTATAATGCCGCGAATTACTCAAGAG
CAB1 Rev + DHA 1603	AAAAGCTCGTGAATACGGCAAGAACGAAGCCCCAGCTTGGAAAAGTACAATTTG
DHA 1603 For + CAB1	TTATTTCAAATTGTACTTTTCCAAGCTGGGGCTTCGTTCTTGCCGTATTC
pGAL Rev + SPE2	GTTAGTCAATTCTTTTATGGTGACAGTCATTATAGTTTTTTCTCCTTGACG
SPE2 For + pGAL	ATACTTTAACGTCAAGGAGAAAAAACTATAATGACTGTCACCATAAAAGAATTG
SPE2 Rev + DHA 1603	AAAAGCTCGTGAATACGGCAAGAACGAAGCGATATTAAATTAGCGTGCTTGC
DHA 1603 For + SPE2	CCCTCCAAGCAAGCACGCTAATTTAATATCGCTTCGTTCTTGCCGTATTC
ILV2 Col Rev	GCGGTATGCTATATGTTGAAAGC
TRR1 Col Rev	CTTTATTGTTCGGAGCAGTGC
ADE4 Col Rev	CATCTTGTCCACGATGTTGTAG

ADE57 Col Rev	CTTGGTGACAAGAACGTGTTC
ADE13 Col Rev	GTTGCTGACATTTCCTTGGAG
ECM31 Col Rev	CATACGCAGTACACATCGACA
CAB1 Col Rev	GCAAGGTTGAAAGTATTGTCGC
SPE2 Col Rev	GCTGATAGTTCGTGGTCAATG
209 UHA For	GAATGTCCGTGGTAATACAATGG
209 UHA Rev + pGAL	CTAATCCGTACTTCAATATAGCAATGAGCCTAGCACATTTTATGGGCCTAAG
pGAL For + 209 UHA	ATAATGTCTTAGGCCCATAAAATGTGCTAGGCTCATTGCTATATTGAAGTAC
TRR1 Rev + 209 DHA	TTGAATACAGAGCAAAAGGATTAGCCATACGTCAGTAGCTGTAATATCAGATG
209 DHA For + TRR1	AATAAAGCATCTGATATTACAGCTACTGACGTATGGCTAATCCTTTTGCTCTG
209 DHA Rev	CTCTATATCGCTGTTGCTTATGG
306 UHA For	GTGACTGTCTCCAAGAATACGAC
306 UHA Rev + pGAL	CTAATCCGTACTTCAATATAGCAATGAGCGCTCCTTCTCCTAACATCAATAACG
pGAL For + 306 UHA	CTGTTCGTTATTGATGTTAGGAGAAGGAGCGCTCATTGCTATATTGAAGTAC
ADE13 Rev + 306 DHA	TTCAGAAACACTGCTTACACTATTCACCAGCGACTGAGTAAGAACCGTTTC
306 DHA For + ADE13	TAAAAATATGAAACGGTTCTTACTCAGTCGCTGGTGAATAGTGTAAGCAGTGTTTC
306 DHA Rev	CAAGAACACCAGACCTCCAAGC
727 UHA For	GACTTGGAAGACCACACTACTCTCC
727 UHA Rev + pGAL	CTAATCCGTACTTCAATATAGCAATGAGCCATAGCAGTGGCGCGGTC
pGAL For + 727 UHA	TTATAGGGAATCGACCGCGCCACTGCTATGGCTCATTGCTATATTGAAGTAC
ECM31 Rev + 727 DHA	ATCAGCAGGCCATGGATAAACTTTCCGTTGGATAAAGACAACAACTCGTG
727 DHA For + ECM31	CTTGGTAGCCCACGAGTTGTTGTCTTTATCCAACGGAAAGTTTATCCATGG
727 DHA Rev	GAGATTCTTGACGTAAAGTGC
1306 UHA For	GGTTTCAAGCCAAATTGTACG
1306 UHA Rev + pGAL	CTAATCCGTACTTCAATATAGCAATGAGCCTTAGGTAGTAACTATACGCAGC
pGAL For + 1306 UHA	GGAGCAGCTGCGTATAGTTACTACCTAAGGCTCATTGCTATATTGAAGTAC
ADE57 Rev + 1306 DHA	TAGCCCACTTCTAGCCAACTTCTAGCCCACCTACAACTCCTCAACAGTATTCTC
1306 DHA For + ADE57	TAGCAAGAGAATACTGTTGAGGAGTTGTAGGTGGGCTAGAAGTTGGCTAGAAG
1306 DHA Rev	GCGCATAGTGCTAGTCTTTTCTCC

* The **red** sequences show the overlapping fragments with neighbour parts, while the **black** sequences show the annealing fragments.
** Plus (+) sign shows the overlapping neighbour parts targeted by the **red** sequences
*** If the overlapping sequences (**red**) show higher affinity than the annealing sequences (**black**) for the same DNA template, only annealing parts should be used first to amplify the target regions.
**** For: forward, Rev: reverse, UHA: upstream homology arm, DHA: downstream homology arm, Col: for colony PCR (coupled with corresponding UHA For)

Table S4: The yeast strains used in the study

Strain	Genotype	Source
LRS6	*MATa, leu2-3, 112::HIS3MX6-GAL1p-ERG19/GAL10p-ERG8; ura3-52::URA3-GAL1p-MvaS^{A110G}/GAL10p-MvaE*; *his3Δ1::hphMX4-GAL1p-ERG12/GAL10p-IDI1; trp1-289::TRP1_GAL1p-CrtE (X.dendrorhous)/GAL10p-ERG20; YPRCdelta15::NatMX-GAL1p-CrtE/GAL10p-CrtE*; *ARS1014::GAL1p-TASY-GFP*; *ARS1622b::GAL1p-MBP-TASY-ERG20*; *ARS1114a::TDH3p-MBP-TASY-ERG20; ARS511b::GAL1p-T5αOH/GAL3-CPR; RKC3::GAL1p-TAT*	Walls et al., 2020
KM1	LRS6, *RKC4*::*GAL1p-TAT*	This study
KM11	KM1, *DPP1Δ*	This study
KM12	KM1, *OAR1Δ*	This study
KM13	KM1, *MDH3Δ*	This study
KM14	KM1, *ACH1Δ*	This study
KM15	KM1, *MLS1Δ*	This study
KM16	KM1, *DIT1Δ*	This study
KM17	KM1, *LPP1Δ*	This study
KM18	KM1, *ISC1Δ*	This study
KM19	KM1, *DTR1Δ*	This study
KM21	KM1, *ARS1603::GAL1p-ILV2*	This study
KM22	KM1, *ARS1603::GAL1p-TRR1*	This study
KM23	KM1, *ARS1603::GAL1p-ADE4*	This study
KM24	KM1, *ARS1603::GAL1p-ADE5,7*	This study
KM25	KM1, *ARS1603::GAL1p-ADE13*	This study
KM26	KM1, *ARS1603::GAL1p-ECM31*	This study
KM27	KM1, *ARS1603::GAL1p-CAB1*	This study
KM28	KM1, *ARS1603::GAL1p-SPE2*	This study
KM31	KM1, *ARS1603*::*ILV2, ARS209::TRR1*	This study
KM32	KM1, *ARS1603*::*ILV2, ARS209::TRR1, ARS306::ADE13, ARS727::ECM31*	This study
KM33	KM1, *ARS1603*::*ILV2, ARS209::TRR1, ARS306::ADE13, ARS727::ECM31, MDH3Δ*	This study
KM34	KM1, *ARS1603*::*ILV2, ARS209::TRR1, ARS306::ADE13, ARS727::ECM31, ARS1531::SPE2, MDH3Δ*	This study
EJ1	KM1, *GAL80Δ*	This study
KMRJ11	EJ1, *DPP1Δ*	This study
KMRJ12	EJ1, *OAR1Δ*	This study
KMRJ13	EJ1, *MDH3Δ*	This study
KMRJ14	EJ1, *ACH1Δ*	This study
KMRJ15	EJ1, *MLS1Δ*	This study
KMRJ16	EJ1, *DIT1Δ*	This study
KMRJ17	EJ1, *LPP1Δ*	This study
KMRJ18	EJ1, *ISC1Δ*	This study
KMRJ19	EJ1, *DTR1Δ*	This study
KMRJ21	EJ1, *ARS1603::GAL1p-ILV2*	This study
KMRJ22	EJ1, *ARS1603::GAL1p-TRR1*	This study
KMRJ23	EJ1, *ARS1603::GAL1p-ADE4*	This study
KMRJ24	EJ1, *ARS1603::GAL1p-ADE5,7*	This study
KMRJ25	EJ1, *ARS1603::GAL1p-ADE13*	This study
KMRJ26	EJ1, *ARS1603::GAL1p-ECM31*	This study
KMRJ27	EJ1, *ARS1603::GAL1p-CAB1*	This study
KMRJ28	EJ1, *ARS1603::GAL1p-SPE2*	This study
KMRJ31	EJ1, *OAR1Δ, DTR1Δ*	This study
KMRJ32	EJ1, *OAR1Δ, DTR1Δ, ARS1603::ADE5,7*	This study
KMRJ33	EJ1, *OAR1Δ, DTR1Δ, ARS1603::ADE5,7, DPPΔ*	This study
KMRJ34	EJ1, *OAR1Δ, DTR1Δ, ARS1603::ADE5,7, DPPΔ, ARS1531::SPE2*	This study

Table S5: The gene candidates predicted by the design algorithms. 17 of them (in bold) were prioritised by *in silico* analyses, maximum potential flux and metabolite interaction maps. The rest of the gene candidates were not used in the experimental studies.

Target Gene*	Design Algorithm	Target Compound	Carbon Source	Intervention
YDL080C (THI3)	OptKnock & OptGene	GGPP	Glucose & Galactose	Knock-out
YOL059W (GPD2)	OptKnock	GGPP	Glucose	Knock-out
YMR303C (ADH2)	OptKnock & OptGene	Acetyl-CoA	Glucose & Galactose	Knock-out
YDR400W (URH1)	OptKnock & OptGene	Acetyl-CoA	Glucose & Galactose	Knock-out
YLR153C (ACS2)	OptKnock & OptGene	Acetyl-CoA & GGPP	Glucose & Galactose	Knock-out
YHR100C (GEP4)	OptKnock	GGPP	Glucose	Knock-out
YKR089C (TGL4)	OptKnock	GGPP	Glucose & Galactose	Knock-out
YML042W (CAT2)	OptKnock & OptForce	Acetyl-CoA	Glucose & Galactose	Knock-out
YKL141W (SDH3)	OptGene	Acetyl-CoA	Galactose	Knock-out
YKL212W (SAC1)	OptKnock	Acetyl-CoA	Galactose	Knock-out
YIL006W (YIA6)	OptKnock & OptGene	Acetyl-CoA	Galactose	Knock-out
YJL097W (PHS1)	OptKnock	Acetyl-CoA	Galactose	Knock-out
YFR044C (DUG1)	OptGene	GGPP	Glucose	Knock-out
YPL268W (PLC1)	OptGene	Acetyl-CoA	Glucose & Galactose	Knock-out
YDR173C (ARG82)	OptGene	GGPP	Glucose & Galactose	Knock-out
YDR196C (CAB5)	OptGene	GGPP	Glucose	Knock-out
YGR209C (TRX2)	OptGene	Acetyl-CoA	Glucose & Galactose	Knock-out
YER086W (ILV1)	OptGene	GGPP	Glucose	Knock-out
YGR015C (EAT1)	OptGene & OptForce	GGPP	Glucose & Galactose	Knock-out
YJL200C (ACO2)	OptGene	GGPP	Glucose	Knock-out
YOR126C (IAH1)	OptGene	Acetyl-CoA	Glucose	Knock-out
YKL215C (OXP1)	OptGene	Acetyl-CoA	Glucose	Knock-out
YPL206C (PGC1)	OptGene	Acetyl-CoA	Glucose & Galactose	Knock-out
YJL005W (CYR1)	OptGene	Acetyl-CoA	Glucose & Galactose	Knock-out
YLL041C (SDH2)	OptGene & OptForce	GGPP	Glucose & Galactose	Knock-out
YER010C	OptGene	GGPP	Glucose & Galactose	Knock-out
YLL041C (SDH2)	OptGene & OptForce	Acetyl-CoA	Glucose & Galactose	Knock-out
YNL065W (AQR1)	OptGene	Acetyl-CoA	Galactose	Knock-out
YLR020C (YEH2)	OptGene	GGPP	Galactose	Knock-out
YLR245C (CDD1)	OptGene	GGPP	Galactose	Knock-out
YPL028W (ERG10)	OptGene & OptForce	Acetyl-CoA	Galactose	Knock-out
YPL092W (SSU1)	OptGene	GGPP	Galactose	Knock-out
YDR001C (NTH1)	OptGene	Acetyl-CoA	Galactose	Knock-out
YOR317W (FAA1)	OptForce	Acetyl-CoA	Glucose	Knock-out
YML059C (NTE1)	OptForce	Acetyl-CoA	Glucose & Galactose	Knock-out
YMR241W (YHM2)	OptForce	Acetyl-CoA	Glucose	Knock-out
YKR009C (FOX2)	OptForce	Acetyl-CoA	Glucose	Knock-out
YOR142W (LSC1)	OptForce	Acetyl-CoA	Galactose	Knock-out
YML120C (NDI1)	OptForce	Acetyl-CoA	Galactose	Knock-out
**YDR284C (*DPP1*)**	OptGene	Acetyl-CoA	Glucose	Knock-out
**YKL055C (*OAR1*)**	OptGene	GGPP	Galactose	Knock-out
**YDL078C (*MDH3*)**	OptKnock & OptForce	Acetyl-CoA	Glucose	Knock-out
**YBL015W (*ACH1*)**	OptKnock & OptGene	Acetyl-CoA	Glucose	Knock-out
**YNL117W (*MLS1*)**	OptKnock	Acetyl-CoA	Galactose	Knock-out
**YDR403W (*DIT1*)**	OptGene	Acetyl-CoA	Glucose	Knock-out
**YDR503C (*LPP1*)**	OptGene	GGPP	Glucose & Galactose	Knock-out
**YER019W (*ISC1*)**	OptGene	Acetyl-CoA	Galactose	Knock-out
**YBR180W (*DTR1*)**	OptGene	GGPP	Glucose	Knock-out
YHR063C (PAN5)	OptForce	Acetyl-CoA	Glucose & Galactose	Overexpression
YGR088W (CTT1)	OptForce	Acetyl-CoA	Glucose & Galactose	Overexpression
YOR184W (SER1)	OptForce	Acetyl-CoA	Glucose & Galactose	Overexpression
YJR024C (MDE1)	OptForce	Acetyl-CoA	Glucose & Galactose	Overexpression
YMR220W** (ERG8)	OptForce	GGPP	Glucose & Galactose	Overexpression

YNR043W** (MVD1)	OptForce	GGPP	Glucose & Galactose	Overexpression
YJL167W** (ERG20)	OptForce	GGPP	Glucose & Galactose	Overexpression
YPL069C** (BTS1)	OptForce	GGPP	Glucose & Galactose	Overexpression
YPL117C** (IDI1)	OptForce	GGPP	Glucose & Galactose	Overexpression
**YMR108W (*ILV2*)**	OptForce	Acetyl-CoA	Galactose	Overexpression
**YDR353W (*TRR1*)**	OptForce	Acetyl-CoA	Galactose	Overexpression
**YMR300C (*ADE4*)**	OptForce	Acetyl-CoA	Glucose & Galactose	Overexpression
**YGL234W (*ADE5,7*)**	OptForce	Acetyl-CoA	Glucose & Galactose	Overexpression
**YLR359W (*ADE13*)**	OptForce	Acetyl-CoA	Glucose & Galactose	Overexpression
**YBR176W (*ECM31*)**	OptForce	Acetyl-CoA	Glucose & Galactose	Overexpression
**YDR531W (*CAB1*)**	OptForce	Acetyl-CoA	Glucose & Galactose	Overexpression
**YOL052C (*SPE2*)**	OptForce	Acetyl-CoA	Glucose & Galactose	Overexpression

* The gene candidates suggested by OptGene or the genes of the related reactions targeted by OptKnock and/or OptForce. Both the gene codes and their scientific names are shown.
** These gene were not used in this study as they were previously integrated into our engineered yeast strain LRS6 [7].
Experimentally tested gene candidates are shown in **bold.**

Table S6: The components of the yeast nitrogen base used in the study

**Component**	**Concentration**
Ammonium sulfate	5 g/L
Biotin	0.002 mg/L
Boric acid	0.5 mg/L
Calcium chloride	0.1 mg/L
Calcium pantothenate	0.4 mg/L
Copper sulfate	0.4 mg/L
Ferric chloride.6H2O	0.2 mg/L
Folic acid	0.002 mg/L
Inositol	2 mg/L
Magnesium sulfate	0.5 g/L
Manganese sulfate.H2O	0.4 mg/L
Niacin	0.4 mg/L
Para-aminobenzoic acid	0.2 mg/L
Potassium iodide	0.1 mg/L
Potassium phosphate monobasic	1 g/L
Pyridoxine HCl	0.4 mg/L
Riboflavin	0.2 mg/L
Sodium chloride	0.1 g/L
Sodium molybdate.2H2O	0.2 mg/L
Thiamine HCl	0.4 mg/L
Zinc sulfate.H2O	0.4 mg/L

Table S7: The components of the complete supplement mixture used in the study

Component	Concentration
Adenine	10mg/L
Arginine HCl	50mg/L
Aspartic Acid	80mg/L
Histidine HCl	20mg/L
Isoleucine	50mg/L
Leucine	100mg/L
Lysine HCl	50mg/L
Methionine	20mg/L
Phenylalanine	50mg/L
Threonine	100mg/L
Tryptophan	50mg/L
Tyrosine	50mg/L
Uracil	20mg/L
Valine	140mg/L

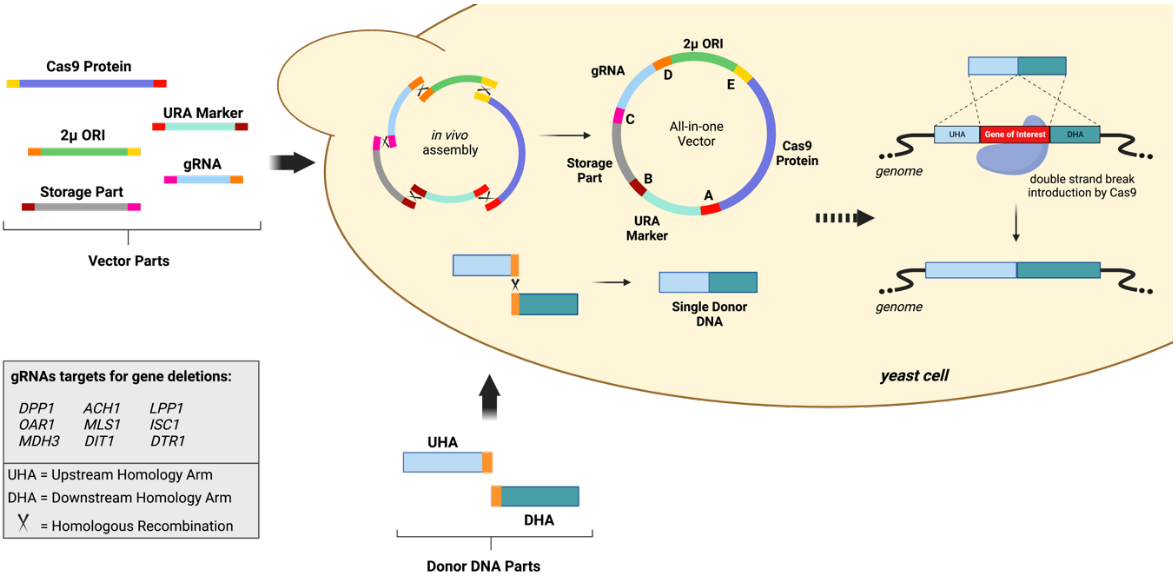

Figure S1: The ACtivE method [8] for gene deletion operates through the following steps: Initially, five CRISPR plasmid parts containing short synthetic overlapping fragments (coloured ends for *in vivo* homologous recombination) are chosen from the ACtivE part repository/toolkit. Next, 500-1000 bp of upstream homology arm (UHA) and downstream homology arm (DHA) flanking the target gene slated for deletion are amplified from the genome. These UHA and DHA regions can share 30-60 bp overlapping fragments, facilitating *in vivo* homologous recombination. In a single-step transformation, all linear parts, including the plasmid components, are co-transformed simultaneously. As a result, the plasmid parts assemble to create a single CRISPR plasmid, while the UHA and DHA assemble to form a single donor DNA using yeast's efficient recombination machinery. The final step involves introducing the donor DNA, consisting of the flanking regions of the target gene, into the yeast genome. This is achieved through homology-directed repair, where the Cas9 induces a double-strand break in the target gene/region, and the donor DNA is inserted through homologous recombination to the yeast genome.

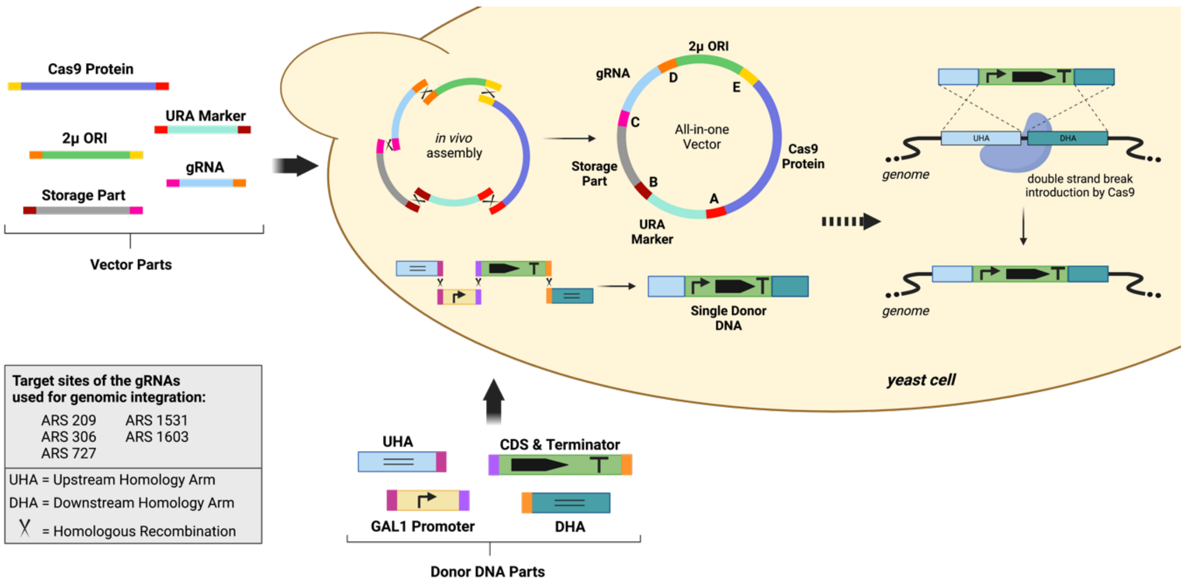

Figure S2: The ACtivE method [8] for genomic integration operates as follows: Initially, linear plasmid parts are carefully selected from the toolkit, and donor DNA parts are amplified, incorporating overlapping fragments between adjacent parts. The donor DNA, essential for genomic integration, consists not only of upstream and downstream homology arms but also includes a promoter and a coding sequence (with terminator). This study employed five out of the eight characterised autonomously replication sequence (ARS) proximal sites available in the ACtivE toolkit [8]. Please refer to Figure S1 for a detailed explanation of the procedure.

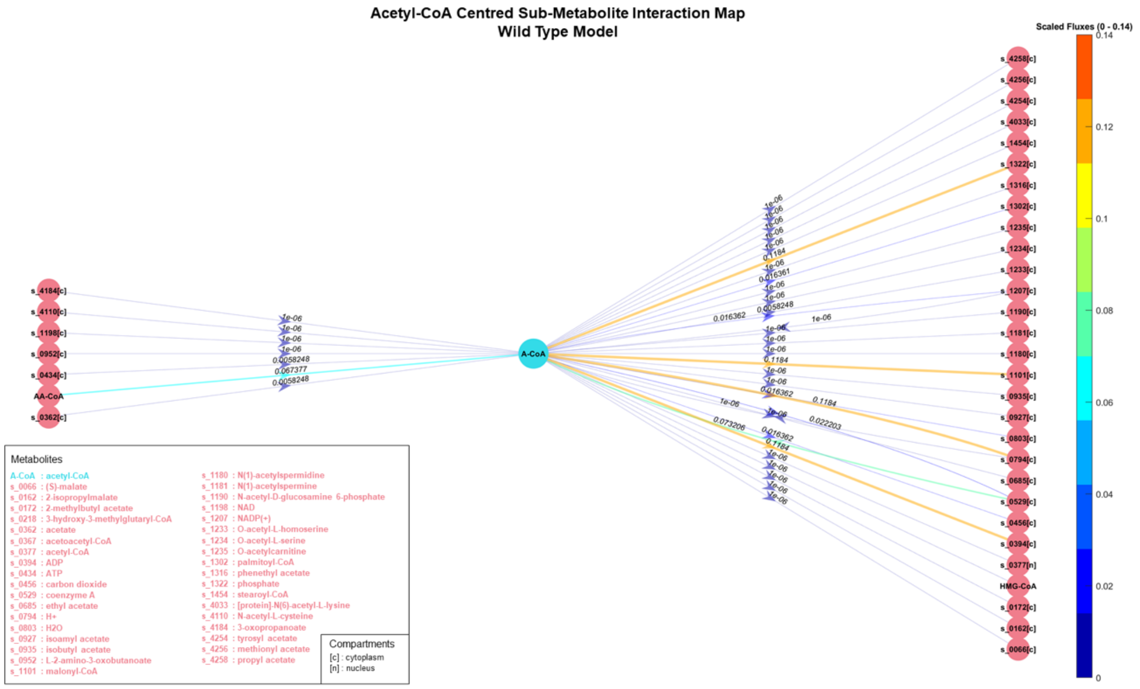

Figure S3: Acetyl-CoA-centred sub-metabolite interaction map of the wild-type model

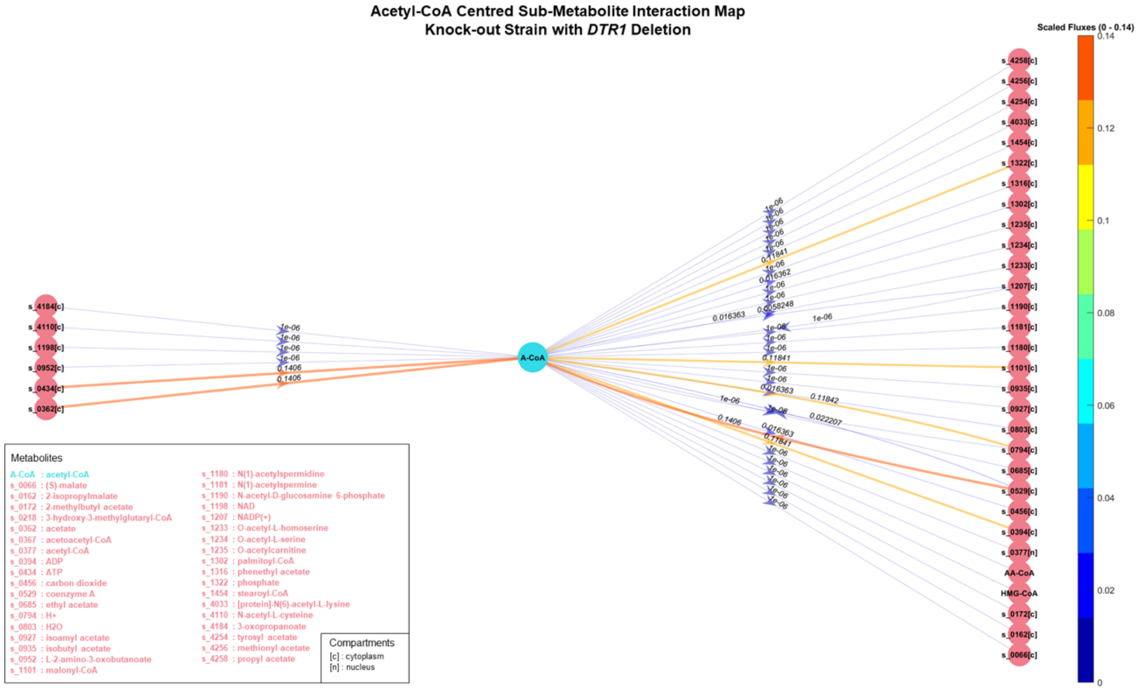

Figure S4: Acetyl-CoA-centred sub-metabolite interaction map of *DTR1* deleted model.

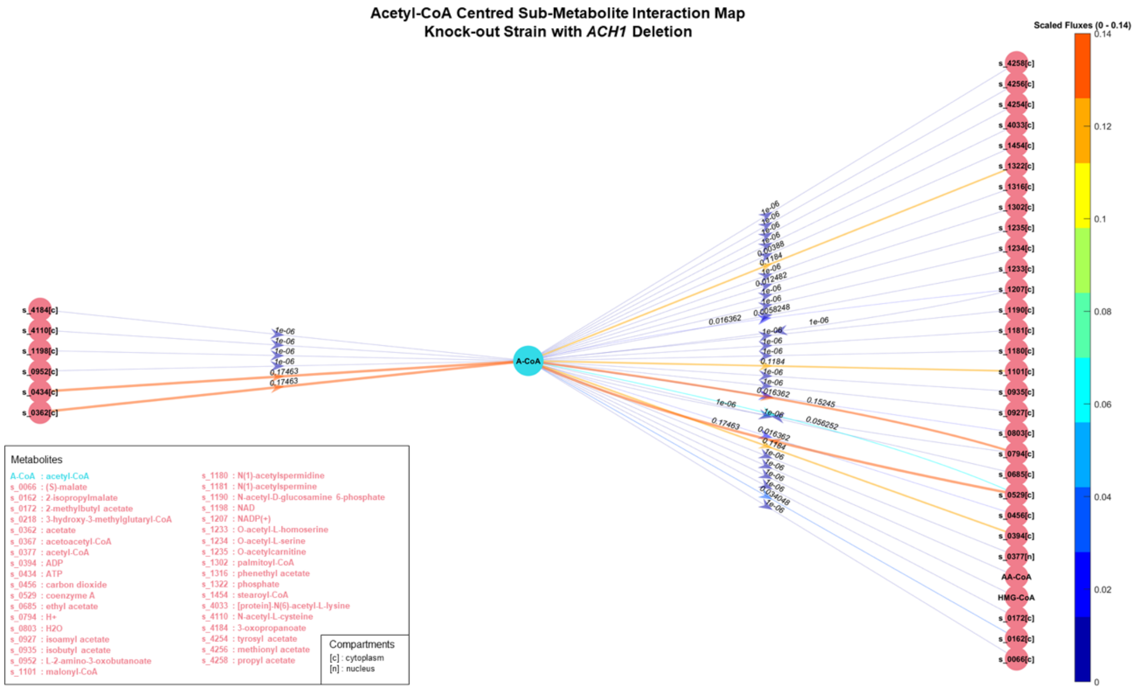

Figure S5: Acetyl-CoA-centred sub-metabolite interaction map of *ACH1* deleted model.

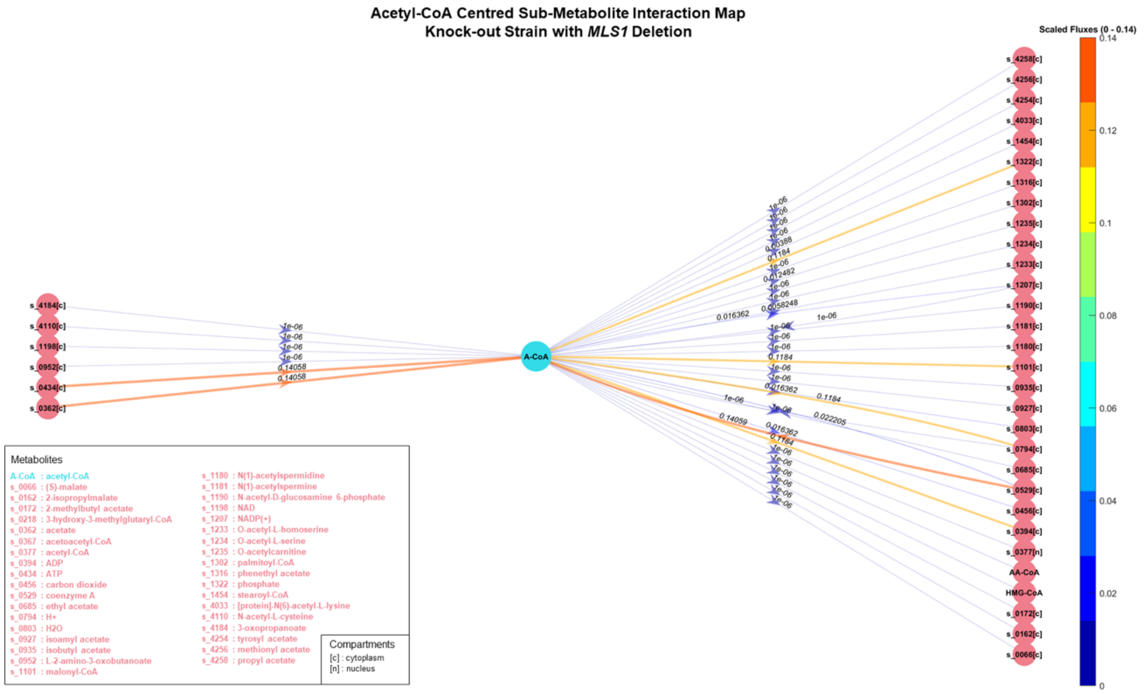

Figure S6: Acetyl-CoA-centred sub-metabolite interaction map of *MLS1* deleted model.

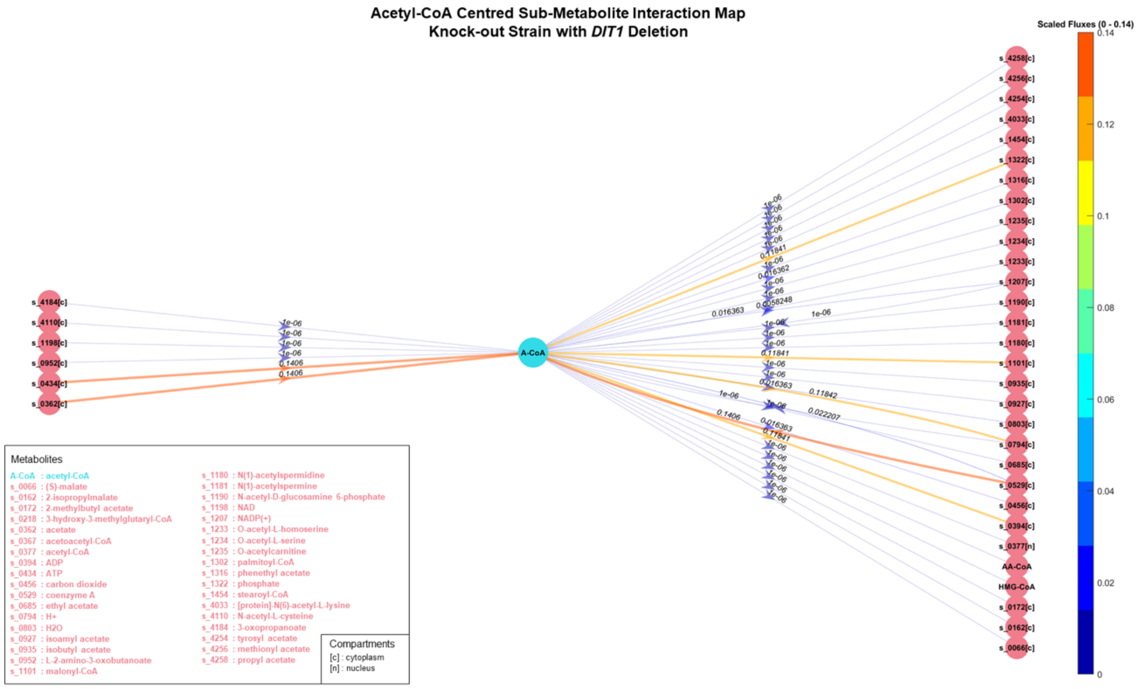

Figure S7: Acetyl-CoA-centred sub-metabolite interaction map of *DIT1* deleted model.

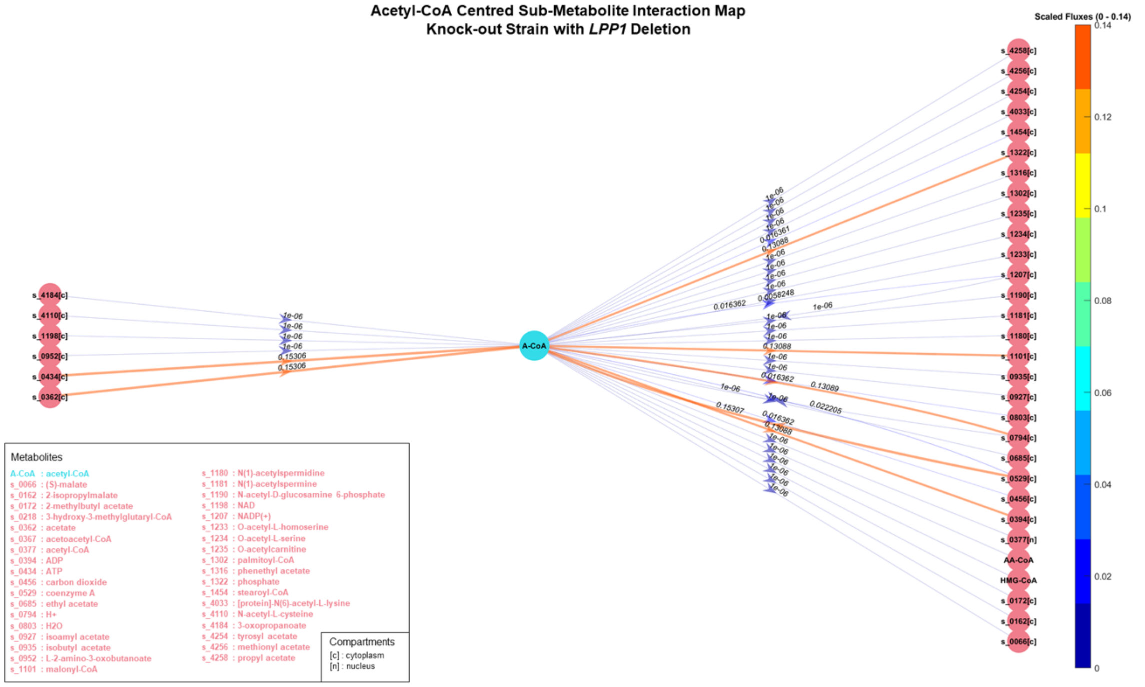

Figure S8: Acetyl-CoA-centred sub-metabolite interaction map of *LPP1* deleted model.

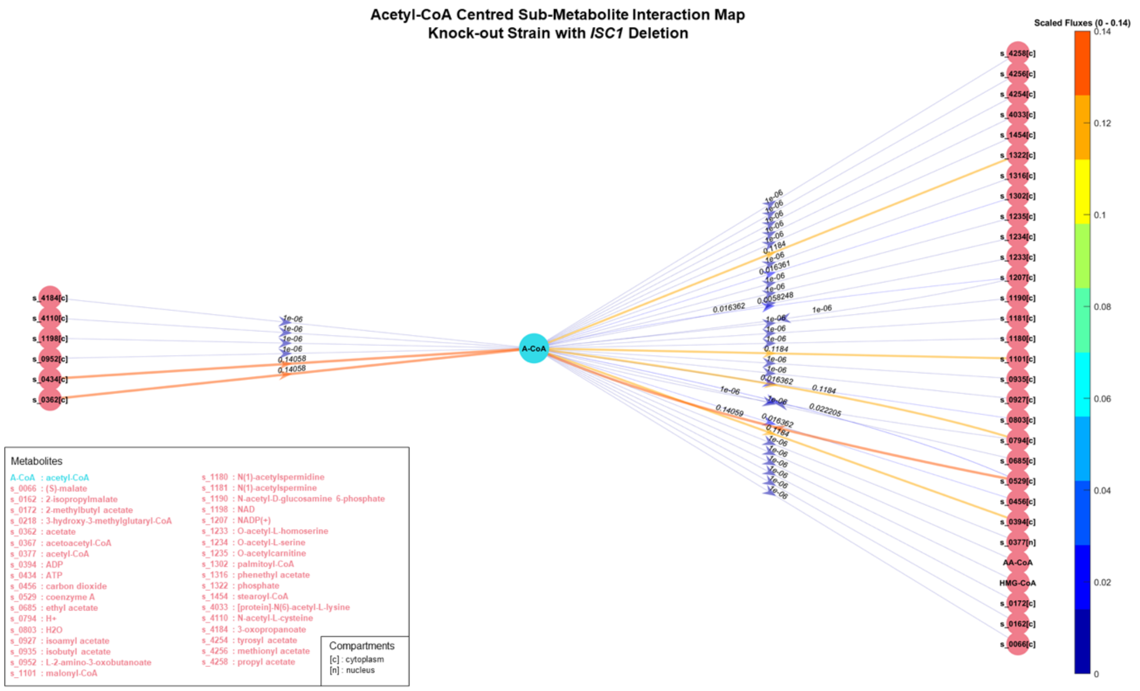

Figure S9: Acetyl-CoA-centred sub-metabolite interaction map of *ISC1* deleted model.

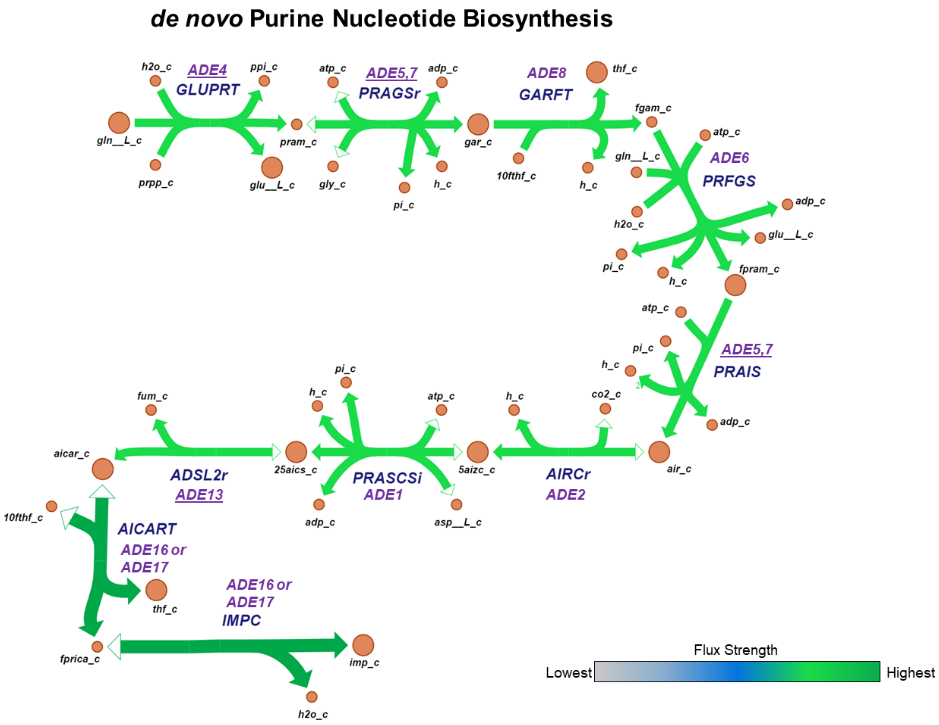

Metabolites

10fthf: 10-Formyltetrahydrofolate
25aics: (S)-2-[5-Amino-1-(5-phospho-D-ribosyl)imidazole-4-carboxamido]succinate
5aizc: 5-amino-1-(5-phospho-D-ribosyl)imidazole-4-carboxylate
adp: ADP
aicar: 5-Amino-1-(5-Phospho-D-ribosyl)imidazole-4-carboxamide
air: 5-amino-1-(5-phospho-D-ribosyl)imidazole
asp_L: L-Aspartate
atp: ATP
fgam: N2-Formyl-N1-(5-phospho-D-ribosyl)glycinamide
fpram: 2-(Formamido)-N1-(5-phospho-D-ribosyl)acetamidine
fprica: 5-Formamido-1-(5-phospho-D-ribosyl)imidazole-4-carboxamide
fum: Fumarate
gar: N1-(5-Phospho-D-ribosyl)glycinamide
gln__L: L-Glutamine
gln_L: L-Glutamine
glu_L: L-Glutamate
gly: Glycine
imp: IMP (C10H11N4O8P)
pi: Phosphate
ppi: Diphosphate
pram: 5-Phospho-beta-D-ribosylamine
prpp: 5-Phospho-alpha-D-ribose 1-diphosphate
thf: 5,6,7,8-Tetrahydrofolate
thf: 5,6,7,8-Tetrahydrofolate

Reactions

ADSL2r: Adenylosuccinate lyase
AICART: Phosphoribosylaminoimidazolecarboxamide formyltransferase
AIRCr: Phosphoribosylaminoimidazole carboxylase
GARFT: Phosphoribosylglycinamide formyltransferase
GLUPRT: Glutamine phosphoribosyldiphosphate amidotransferase
IMPC: IMP cyclohydrolase
PRAGSr: Phosphoribosylglycinamide synthase
PRAIS: Phosphoribosylaminoimidazole synthase
PRASCSi: Phosphoribosylaminoimidazolesuccinocarboxamide synthase
PRFGS: Phosphoribosylformylglycinamidine synthase

Compartments

_c: cytosol

Figure S10: *de novo* purine nucleotide biosynthesis pathway. The orange circles represent the metabolites involved in the reactions, and the genes are shown in purple. The underlined genes show the overexpressed genes. The arrows indicate the direction of the fluxes.

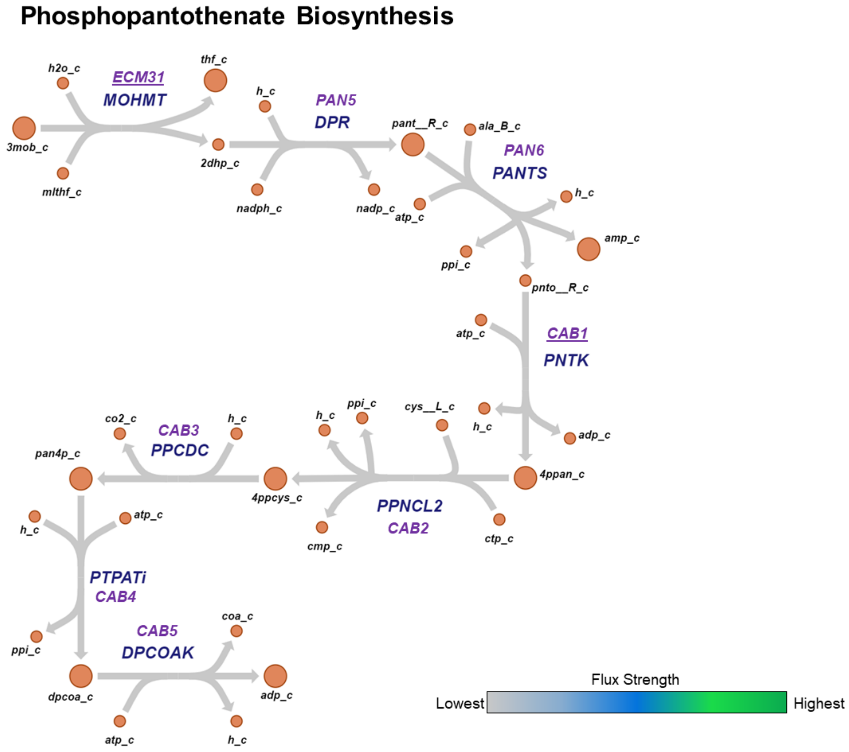

Metabolites

2dhp: 2-Dehydropantoate
3mob: 3-Methyl-2-oxobutanoate
4ppan: D-4'-Phosphopantothenate
4ppcys: N-((R)-4-Phosphopantothenoyl)-L-cysteine
ala_B: Beta-Alanine
amp: AMP
atp: ATP
cmp: CMP
coa: Coenzyme A
ctp: CTP
cys__L: L-Cysteine
dpcoa: Dephospho-CoA
mlthf: 5,10-Methylenetetrahydrofolate
nadp: Nicotinamide adenine dinucleotide phosphate
nadph: Nicotinamide adenine dinucleotide phosphate - reduced
pan4p: Pantetheine 4'-phosphate
pant_R: (R)-Pantoate
pnto_R: (R)-Pantothenate
ppi: Diphosphate
thf: 5,6,7,8-Tetrahydrofolate

Reactions

DPCOAK: Dephospho-CoA kinase
DPR: 2-dehydropantoate 2-reductase
MOHMT: 3-methyl-2-oxobutanoate hydroxymethyltransferase
PANTS: Pantothenate synthase
PNTK: Pantothenate kinase
PPCDC: Phosphopantothenoylcysteine decarboxylase
PPNCL: Phosphopantothenate-cysteine ligase
PTPATi: Pantetheine-phosphate adenylyltransferase

Compartments

_c: cytosol

Figure S11: Phosphopantothenate biosynthesis pathway. The orange circles represent the metabolites involved in the reactions, and the genes are shown in purple. The underlined genes show the overexpressed genes. The arrows indicate the direction of the fluxes.

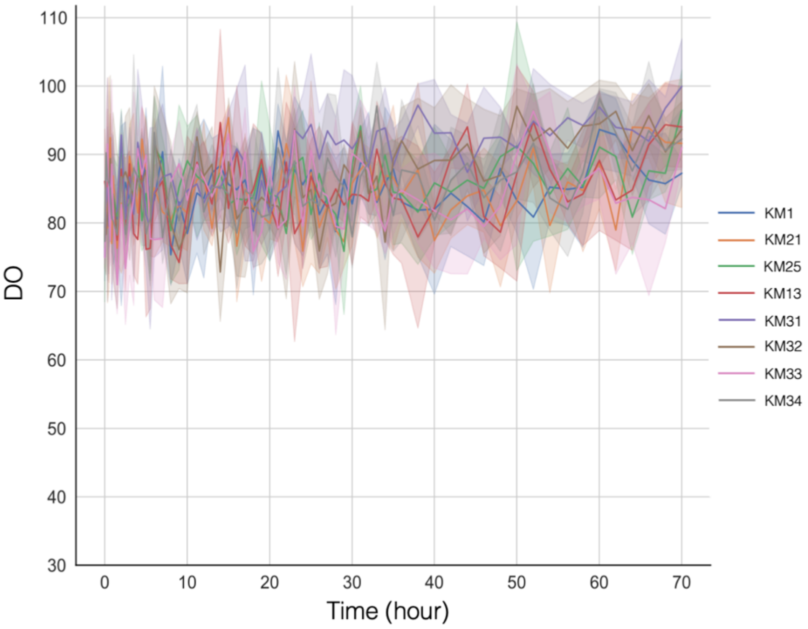

Figure S12: Dissolved oxygen (DO) concentrations of three-day cultures of KM1-derived strains measured by the BioLector microbioreactor system in the galactose-containing CSM.

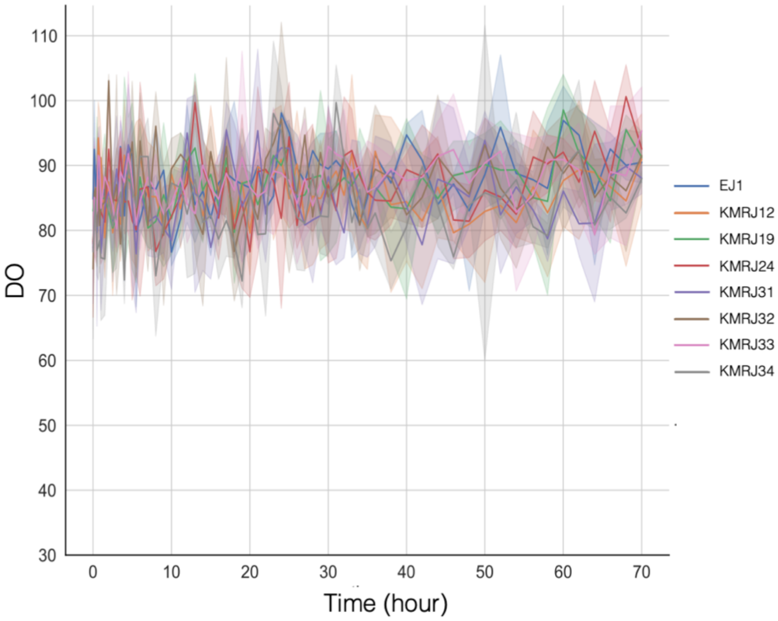

Figure S13: Dissolved oxygen (DO) concentrations of three-day cultures of EJ1-derived strains measured by the BioLector microbioreactor system in the glucose-containing CSM.

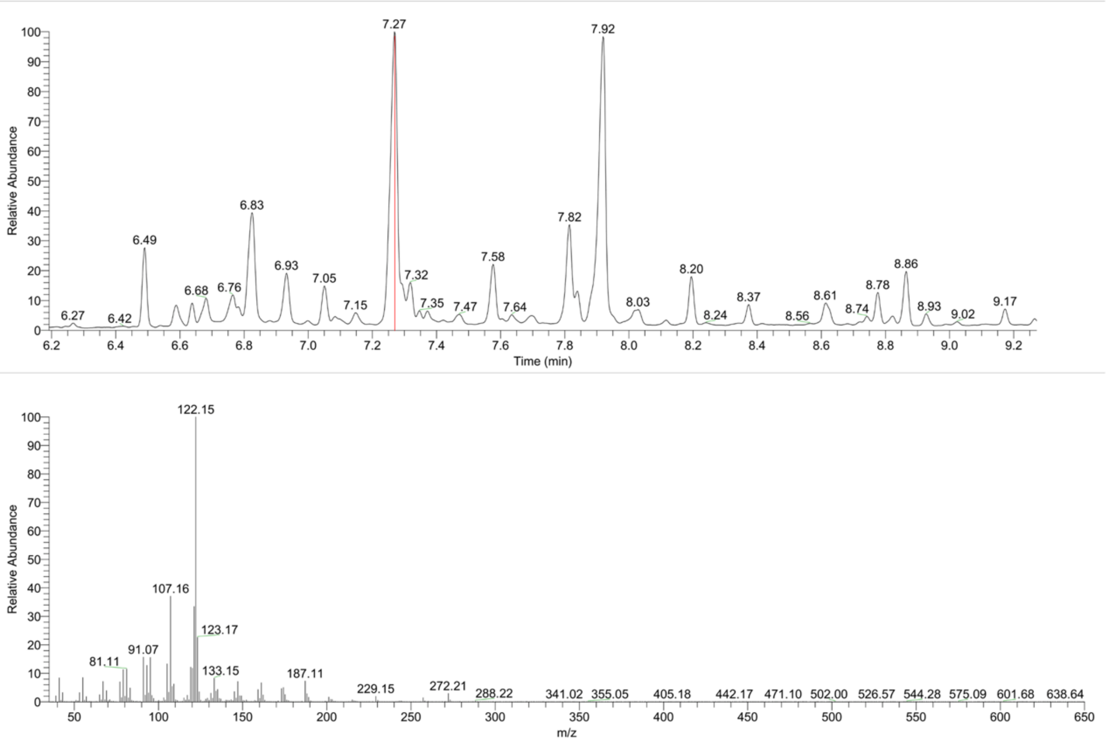

Figure S14: Gas chromatography shows the compounds' peaks produced by KM32 and the mass spectrum of taxadiene. The retention time of the taxadiene peak was at 7.27th minutes.

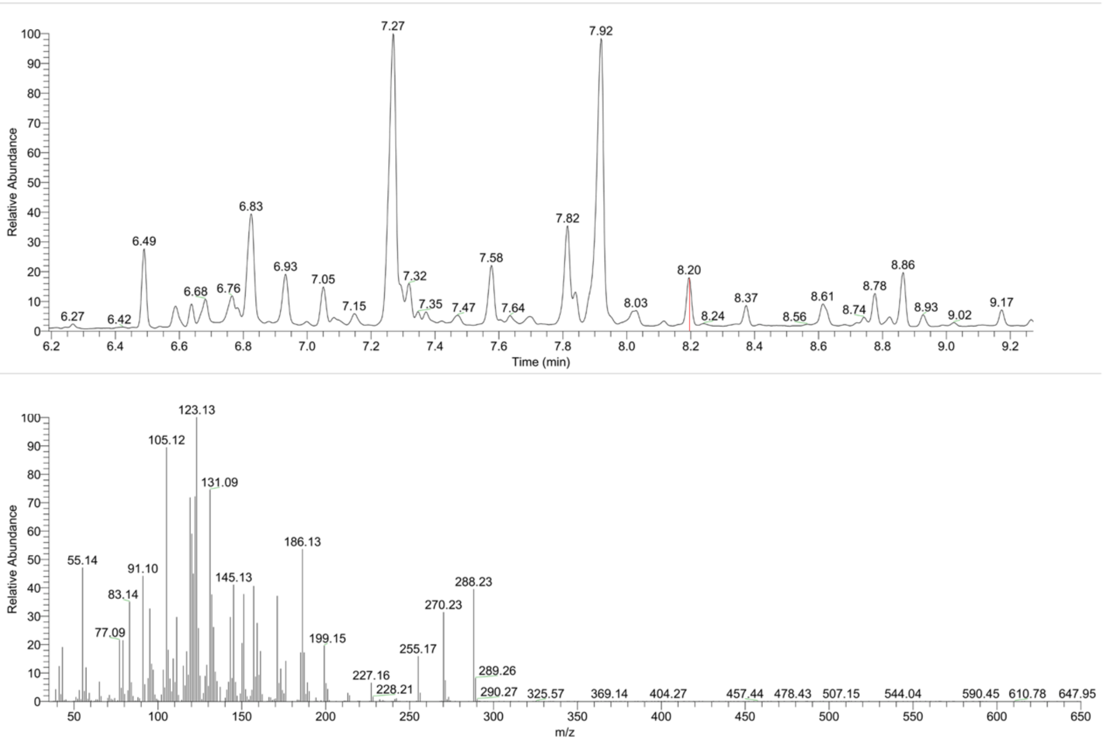

Figure S15: Gas chromatography shows the compounds' peaks produced by KM32 and the mass spectrum of T5α-ol. The retention time of the T5α-ol peak was at 8.20th minutes.

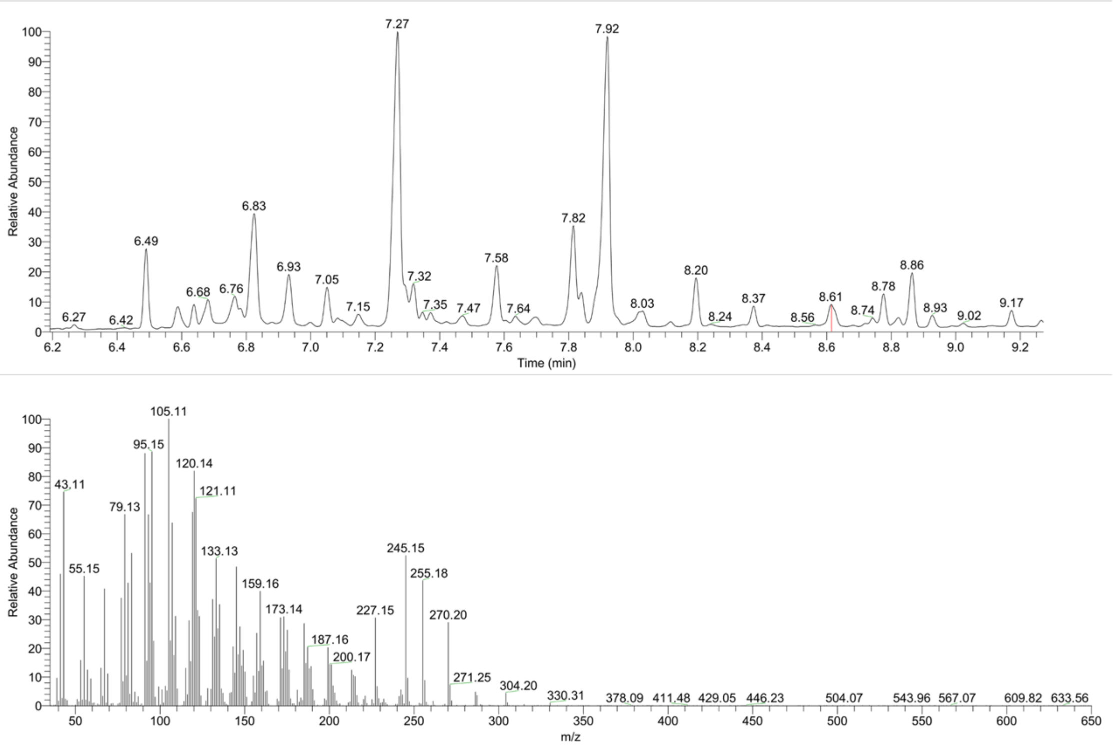

Figure S16: Gas chromatography shows the compounds' peaks produced by KM32 and the mass spectrum of T5αAc. The retention time of the T5αAc peak was at 8.61st minutes. Note that T5αAc might coelute with taxadiendiol, therefore, their peaks might be overlapped.

References

1. Burgard AP, Pharkya P, Maranas CD (2003) OptKnock: A Bilevel Programming Framework for Identifying Gene Knockout Strategies for Microbial Strain Optimization. Biotechnol Bioeng 84:647–657. https://doi.org/10.1002/BIT.10803
2. Ranganathan S, Suthers PF, Maranas CD (2010) OptForce: An Optimization Procedure for Identifying All Genetic Manipulations Leading to Targeted Overproductions. PLOS Comput Biol 6:e1000744. https://doi.org/10.1371/JOURNAL.PCBI.1000744
3. Patil KR, Rocha I, Förster J, Nielsen J (2005) Evolutionary programming as a platform for in silico metabolic engineering. BMC Bioinformatics 6:1–12. https://doi.org/10.1186/1471-2105-6-308/
4. Gudmundsson S, Thiele I (2010) Computationally efficient flux variability analysis. BMC Bioinformatics 11:1–3. https://doi.org/10.1186/1471-2105-11-489
5. Kocabaş K, Arif A, Uddin R, Çakır T (2022) Dual transcriptome based reconstruction of Salmonella-human integrated metabolic network to screen potential drug targets. PLoS One 17:. https://doi.org/10.1371/JOURNAL.PONE.0268889
6. openCOBRA - createMetIntrcNetwork COBRA.tutorials/visualization/createMetIntrcNetworkTutorial at master · opencobra/COBRA.tutorials · GitHub. https://github.com/opencobra/COBRA.tutorials/tree/master/visualization/createMetIntrcNetworkTutorial. Accessed 26 Feb 2023

7. Walls LE, Malcı K, Nowrouzi B, et al (2020) Optimizing the biosynthesis of oxygenated and acetylated Taxol precursors in Saccharomyces cerevisiae using advanced bioprocessing strategies. Biotechnol Bioeng 118:279–293. https://doi.org/10.1002/BIT.27569
8. Malcı K, Jonguitud-Borrego N, Van Der Straten Waillet H, et al (2022) ACtivE: Assembly and CRISPR-Targeted in Vivo Editing for Yeast Genome Engineering Using Minimum Reagents and Time. ACS Synth Biol 2022:3629–3643. https://doi.org/10.1021/acssynbio.2c00175
